# Association Between Coronary Artery Disease and MicroRNA: Literature Review and Clinical Perspective

**DOI:** 10.7759/cureus.1188

**Published:** 2017-04-23

**Authors:** Rehan Malik, Raja S Mushtaque, Usman A Siddiqui, Adnan Younus, Muhammad A Aziz, Choudhry Humayun, Kanaan Mansoor, Muhammad A Latif, Salman Waheed, Salman Assad, Idrees Khan, Syed M Bukhari, Daniel DelCampo, Ali Adus, Swetha Gannarapu

**Affiliations:** 1 Internal Medicine, Mount Sinai Medical Center, Miami Beach, Florida,; 2 Center for Healthcare Advancement & Outcomes Research, Baptist Health Medical Group; 3 Weiss Memorial Hospital, Internal Medicine, University of Illinois at Chicago; 4 Cardiology-University of Kansas Hospital & Medical Center, University of Kansas Hospital & Medical Center; 5 Department of Medicine, Shifa Tameer-e-Millat University, Islamabad, Pakistan; 6 UPMC Mckeesport Internal Medicine Department, University of Pittsburgh Medical Center

**Keywords:** mirna, coronary artery disease, association

## Abstract

**Background:**

Until recently, circulating micro-RNAs (miRNAs) have attracted major interest as novel biomarkers for the early diagnosis of coronary artery disease (CAD). This review article summarizes the available evidence on the correlation of micro-RNAs with both the clinical and subclinical coronary artery disease and highlights the necessity for exploring miRNAs as a potential diagnostic and prognostic biomarker of early CAD in an adult population.

**Methods:**

A systematic literature analysis and retrieval online systems Public/Publisher MEDLINE/ Excerpta Medica Database /Medical Literature Analysis and Retrieval System Online,(PUBMED/EMBASE/MEDLINE) search were conducted for relevant information. Search was limited to the articles published in English language and conducted on humans, January 2000 onwards. We excluded studies of heart surgery, coronary artery bypass grafting (CABG), angioplasty and heart transplant. Eighteen studies met the inclusion criteria.

**Results:**

Seven out of 18 studies were multivariate, i.e. adjusted for age, gender, body mass index (BMI), smoking, hypertension, diabetes, and blood lipid profiles, while the remaining twelve studies were univariate analysis. Different sources of miRNAs were used, i.e. plasma/serum, microparticles, whole blood, platelets, blood mononuclear intimal and endothelial progenitor cells were investigated. Fourteen out of 18 studies showed up-regulation of different miRNA in CAD patients and in vulnerable plaque disease. Four out of 18 studies showed both the up-regulation and down-regulation of miRNA in the population, while only three studies showed down-regulation of miRNA. Various sources and types of miRNA were used in each study.

**Conclusion:**

This review gives an extensive overview of up-regulation and down-regulation of miRNA in CAD and non-CAD patients. The pattern of miRNA regulation with respect to CAD/non-CAD study subjects varies across individual studies and different parameters, which could be the possible reason for this aberrancy. We suggest further trials be conducted in future for highlighting the role of miRNA in CAD, which may improve both the diagnostic and therapeutic approaches to stratifying CAD burden in the general population.

## Introduction

Heart disease is the leading cause of death for both males and females with more than half of the deaths reported in 2009 in males [1]. Coronary heart disease is the most common type of heart disease with 370,000 annual deaths, i.e. each minute someone in the United States dies from a heart disease-related event [2]. Coronary heart disease alone each year costs the United States $108.9 billion, which includes the cost of health care services, medications, and lost productivity [3]. The total coronary artery disease (CAD) prevalence is 6.4% in US adults, which is expected to increase approximately 18% by 2030 [4]. Most individuals aged over 60 years have progressively enlarged deposits of calcium mineral in the plaques in their major arteries [5]. As atherosclerosis infiltrates the arterial wall long before it causes vessel obstruction and produces symptoms, earlier identification of this process should be part of risk prediction [6]. As such, there is a lack of cost-effective and specific biomarkers for the early clinical diagnosis and prognosis of CAD, and there is an immense clinical demand for specific and reliable non-invasive biomarkers for CAD.

With over 1900 MicroRNA (miRNAs) discovered in humans to date, many of them have already been implicated in common human disorders. However, the pattern among the miRNA-disease association remains largely unclear for most diseases. Until recently, circulating micro-RNAs (miRNAs) have attracted major interest as novel biomarkers for the early diagnosis of CAD [7]. MiRNAs are a class of small (~22 nucleotides long), highly specific, endogenous, single-stranded, non-coding RNAs that regulate the expression of target genes by binding to the 39 untranslated regions and degrading or inhibiting the translation of messenger ribonucleic acid (RNA) (mRNAs) [8]. Studies have shown miRNAs' involvement in the timing of cell death and cell proliferation, hematopoiesis, and other normal cellular homeostasis [9-10]. Various miRNAs are expressed in a tissue-specific manner and thus may regulate tissue-specific functions. This review article summarizes the available evidence correlating micro-RNA, clinical and subclinical CAD and further highlights the necessity for exploring the potential of micro-RNAs as useful diagnostic and prognostic biomarkers for early CAD in the adult population.

## Materials and methods

**Figure 1:**
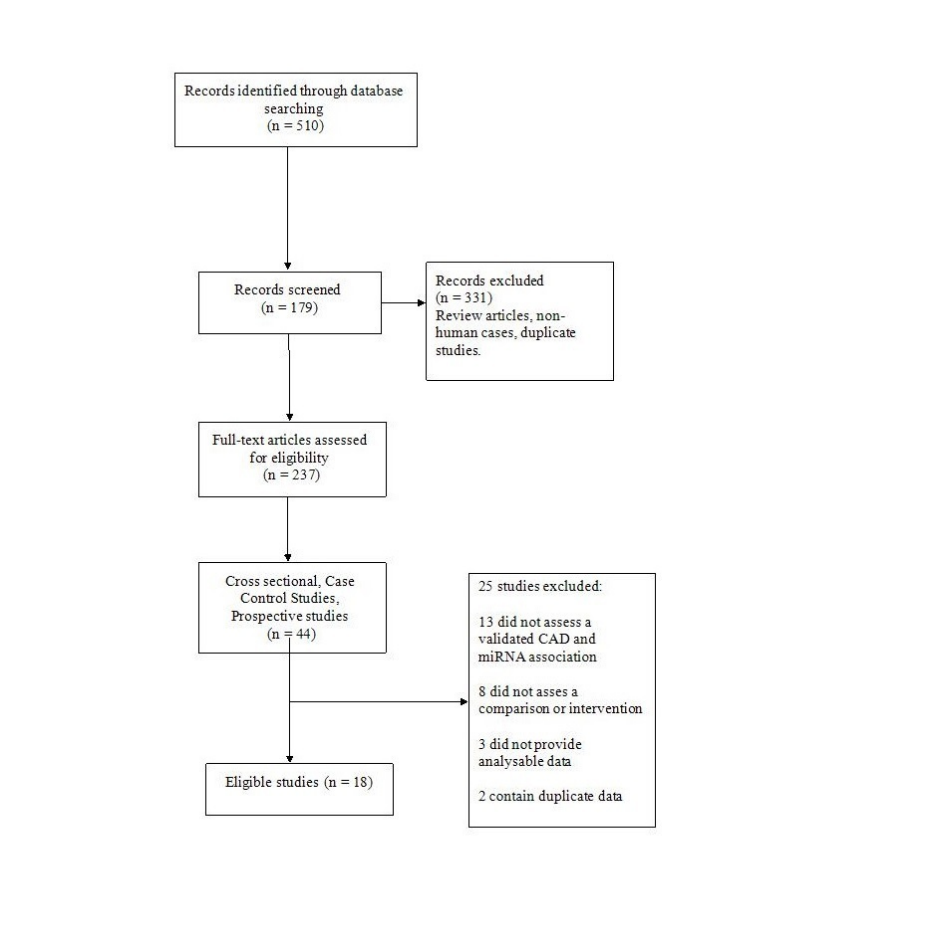
Detailed literature analysis- CAD and miRNA association

A computerized search of the Public/Publisher MEDLINE/ Excerpta Medica Database /Medical Literature Analysis and Retrieval System Online/Excerpta Medica Database (PubMed/Medline/EMBASE) database was done with the keywords and medical subject headings (MESH) terms such as “micro RNA,” “coronary artery disease,” “cardiovascular disease (CVD),” “Subclinical CVD,” “coronary artery calcium and micro RNA,” "miRNA and high sensitivity C- reactive protein (hs-CRP),” “miRNA and coronary intimal thickness,” and “miRNA and pulse wave velocity.” We included all the literature that was published from January 1, 2000, until January 1, 2017. The search was limited to articles published in the English language. Included studies were cross-sectional, case-control or prospective in design and conducted in adult populations (Figure 1). CAD subjects diagnosed by symptoms, imaging, cardiac enzymes, electrocardiogram (EKG), diagnostic angiography or stress testing were included. We excluded studies with CAD patients who have had heart surgery, coronary artery bypass graft (CABG), angioplasty, and heart transplant. We also examined the references of all studies from the initial search for additional references. Demographic data was extracted from each study and results were collaborated into tables.

## Results

A total of 18 clinical studies has been included in the review after a thorough analysis of the literature. Overall, there were 1720 subjects. The majority of the studies included were done in China, which was 11 in number, while two studies from Japan, one from the USA, one from Norway, one from Netherlands, one from the UK and one from Germany were included (Table 1). All the studies had the same outcome, which was CAD. Studies were further divided into two groups of microRNA up-regulation and down-regulation. There were four studies that checked for both up-regulation and down-regulation of microRNA. 

**Table 1:** Demographics table A :Age, M: Male, CAD: Coronary Artery Disease, AP: Atherosclerotic Plaque, Pre: Premature, UA: Unstable Angina, HLD: Hyperlipidemia, ACS: Acute Coronary Syndrome, ASCL: Atherosclerosis, VC: Validation Cohort, UAP: Unstable Angina Pectoris, SAP: Stable Angina Pectoris, AO : Arteriosclerosis Obliterans

Serial#	Author, Year, Study Population	Study Population Characteristics	Country	Type of Study	Outcome
1	Diehl, et al.,2011 [8]; CAD (n=5), ACS (n=5)	CAD: A: 76±2; M: 3 (60%) ACS: A: 66±4; M 5 (100%)	China	Case Control	CAD
2	Sayed, et al., 2015 [11]; CAD (n=65), Non-CAD (n=32)	CAD: A: 53 (49–57); M: 38 (58.4%) Non-CAD: A: 53 (49–57); M: 16(50%)	China	Case Control	CAD
3	Sayed, et al., 2015 [12]; CAD (n=37), Non-CAD (n=20)	CAD: A: 72.97 ± 4.28; M: 18 (67.5%) Non-CAD: A: 71.7 ± 5.2, M: 10(50%)	China	Case Control	CAD
4	Zhou, et al., 2015 [13]; CAD (n=67),Non-CAD (n=67)	CAD: A 64.70± 6.79 M: 43 (64.2%) Non-CAD: A: 63.69± 5.96 M: 32 (47.8%)	China	Case Control	CAD
5	Liu, et al., 2015 [14];CAD (n=90), Non-CAD (n=70)	Not Given	China	Case Control	CAD
6	Han, et al., 2015 [15]; CAD (n=32), Non-CAD (n=20)	CAD A: 67±11; M: 32 (100%) Non-CAD: A 62±8; M: 20 (100%)	China	Case Control	CAD
7	Zhu, et al., 2014 [16];CAD (n=56), Non-CAD (n=54)	Not Given	China	Case Control	CAD
8	Li, et al., 2013 [17]; Pre-CAD(n=12), Non-CAD (n=12)	Age/Sex matched in pre CAD and Non CAD.	China	Case Control	CAD
9	Ren, et al., 2013 [18]; CAD (UA) (n=45), Non-CAD (n=37)	CAD (UA): A: 63±12; M: 25 (55.5%) Non-CAD: 59±6, M: 22 (59.4%)	China	Case Control	CAD
10	Guo, et al., 2012 [19]; HLD+CAD (n=155), non-HLD (n=100)	HLD+CAD: A: 65.3 ± 11.0; M: 105 (67.7%) Non-CAD: A 63.0 ± 10.7; M: 35 (70%)	China	Case Control	CAD
11	Weber, et al., 2011 [20]; CAD (n=10), Non-CAD (n=15)	Age and sex matched	USA	Case Control	CAD
12	Sondermeijer, et al., 2011 [21]; Pre CAD (n=40), Non-CAD (n=40) ASCL (n=27), family(n=40)	Age matched and males only; controls > 20 yr younger, sex unknown	Netherlands	Case Control	CAD
13	Fichtlscherer, et al., 2011 [22];CAD (n=67), Non-CAD (n=31)	Cohort; CAD: A: 67.69 ± 11.07; M: 25 (69.4%) Non-CAD: A: 32.18 ± 8.78Male: 6 (35.3%); VC; CAD: A: 68.06 ± 9.66; M: 21 (68%) Non-CAD: A: 39.28 ± 17.52; M: 5: (36%)	Germany	Case Control	CAD
14	Taurino, et al., 2010 [23]; CAD (n=12), Non-CAD (n=12)	CAD: A: 66±11; M: 12 (100%) Non-CAD: A: 597; M: 12 (100%)	UK	Case Control	CAD
15	Hoesktra, et al., 2010 [24]; UAP (n=25), SAP (n=25), Non- CAD (n=20)	Age, sex, ethnically, smoking matched	Norway	Case Control	CAD
16	Takahashi, et al., 2010 [25]; Stable CAD (n=66), Non-CAD (n=33)	CAD: A: 66.2±9.5, M: 52 (79%) Non-CAD: A: 64.2±10.3, M: 26 (79%)	Japan	Case Control	CAD
17	Li, et al., 2010 [26]; AO (n=104), Non-AO (n=105)	Age Matched	China	Case Control	CAD
18	Minami, et al., 2009 [27]; Stable CAD (n=44), Non-CAD (n=22)	CAD: A 66.1±12.8; M: 36 (82%). Non-CAD: A: 64.5 ± 8.5, M: 17 (77%)	Japan	Case Control	CAD

### Up-regulation and microRNA 

A total of 15 studies reported up-regulation of micro RNAs in patients with CAD. Ten studies out of the 15 used plasma as the source while two studies used peripheral blood mononuclear cells as the source, one study used microparticles (MP) from plasma, one study used endothelial progenitor cells (EPC) and one study used platelets. The majority of the studies employed quantitative reverse transcription polymerase chain reaction (QRT-PCR) for mircoRNA analysis while only one study employed Kyoto encyclopedia of genes and genomes (KEGG) method for microRNA analysis (Li, et al. 2013). All studies reported that there is up-regulation of specific miRNA in relationship to CAD. Univariate analysis was done in nine of the 15 studies, while remaining studies adjusted for age, sex, high-density lipoprotein (HDL), low-density lipoprotein (LDL), aspartate aminotransferase (AST), alanine aminotransferase (ALT), hypertension HTN etc in their analysis (Table 2).

**Table 2:** MicroRNA up-regulated studies QRT-PCR: Quantitative Reverse Transcription Polymerase Chain Reaction, CAD: Coronary Artery Disease, DM: Diabetes Mellitus, HTN: Hypertension, TC: Total Cholesterol, TAG Triacylglycerols, HDL-c: High Density Lipoprotein Cholesterol, LDL-c: Low Density Lipoprotein Cholesterol, AST: Aspartate Aminotransferase, ALT: Alanine Transaminase, LVEF: Left Ventricle Ejection Fraction, CK-MB: Creatine Kinase Myocardial B fraction, LDH: Lactate Dehydrogenase, BMI: Body Mass Index, HLD : Hyperlipidemia, ACS: Acute Coronary Syndrome, KEGG: Kyoto Engenomes, SMCS: Smooth Muscle Cells, HBA1C: Hemoglobin A1C, CRP: C-Reactive Protein, TLR: Toll Like Receptor, AO: Arteriosclerosis Obliterans. EP: Endothetial Progenitor Cells, MP- Microparticles

Serial #	Name of Author; Year of Study	MicroRNA (miR or miRNA)	MicroRNA: Regulation	MicroRNA Analysis	Source	Strength of Association (Odds ratio, Relative Risk or Regression Analysis)	Comments
1	Diehl, et al. 2012 [8]	miR 19 miR 21 miR 146 miR 155 miR 223	Up-regulated: miR 19 miR 21 miR 146 miR 155 miR 223	QRT-PCR	MP from plasma	ACS vs CAD miR 21 P=0.042 miR 146a P=0.003	Univariate Analysis
2	Sayed, et al., 2015 [11]	miR149 miR 424 miR 765	Up-regulated: miR 765	QRT-PCR	Serum/ Plasma	Non-CAD vs. CAD (Adjusted) miR 149 95% CI 0.894 to 0.983 p= <0.0001 miR 424 95% CI 0.863 to 0.975 p = <0.0001 miR 765 95% CI 0.939 to 0.996 p=0.0001	Adjusted for age, gender, TC, TAG, HDL-C, LDL-C, systolic blood pressure, diastolic blood pressure, AST, ALT, creatinine, LVEF, DM, smoking, HTN and medications
3	Sayed, et al., 2015 [12]	miR 149 miR 765	Up-regulated: miR 765	QRT-PCR	Serum/ Plasma	Stable/unstable CAD vs Non-CAD (Adjusted) miR 765 p=< 0.001	Adjusted for subjects with similar age, gender, total cholesterol, total glyceride, HDL, LDL, systolic blood pressure, diastolic blood pressure, AST, ALT, creatinine, cardiac troponinI, CK-MB, LDH, LVEF, DM, smoking, HTN, and medications
4	Zhou, et al., 2015 [13]	miR206 miR574/5p	Up-regulated: miR 206 miR 574/5p	QRT-PCR	Plasma	CAD vs Non-CAD (Adjusted) miR 206 –95% CI: (0.508-0.706) miR 574/5p – 95% CI: (0.609- 0.787)	Multivariate analysis (no significant differences between two groups including HTN, DM, smoking history, age, gender, HDL-C, TAG, LDL-C and TC)
5	Liu, et al., 2015 [14]	miR 2861 miR 3135b miR191/3p miR133a/3p miR1229/5p miR134 miR3679/5p miR223	Up-regulated: miR 133a/3p miR 134 miR 191/3p miR 223 miR 1229/5p miR 2861 miR 3135b miR 3679/5p	QRT-PCR	Serum/ Plasma	Unadjusted miR 133a/3p 95% CI 0.55-0.82 p = 0.0096 miR 134 95% CI 0.54-0.82 p= 0.015 miR 191/3p 95% CI 0.58-0.83p= 0.0046 miR 223 95% CI 0.47-0.74p= 0.13 miR 1229-5p 95% CI 0.54-0.83 p= 0.015 miR 286195% CI 0.61-0.87 p= <0.001 miR 3135b95% CI 0.61-0.86 p= <0.001 miR 3679/5p95% CI 0.49-0.79 p = 0.06	Univariate Analysis Matched for sex, DM, and age. A key feature in vasculature calcification is the osteogenic transition of SMCs miR 2861 might function as an enhancer of osteogenic differentiation of SMCs. The increased circulating miR 2861 level may reflect CAC progression.
6	Han, et al., 2015 [15]	miR 21a miR 23a miR 34a	Up-regulated: miR 21 miR 23a miR 34a	QRT-PCR	Serum/ Plasma	CAD vs Non-CAD miR-34a, miR-21, and miR-23a that are differentially expressed in CAD plasma p=<0.01	Univariate Analysis
7	Li, et al., 2013 [16]	miR 526b	Up-regulated: miR 526b	MiRNA by KEGG	Serum/ Plasma	Pre CAD vs Non-CAD (Adjusted) miR 526b p=0.00072, p=0.003215	Univariate Analysis
8	Ren, et al., 2013 [18]	miR 21 miR 25 miR 92a miR 106b miR 126 miR 451 miR 590/5p	Up-regulated: miR 21 miR 25 miR 92a miR 106b miR 126 miR 451 miR 590/5p	QRT-PCR	Serum/ Plasma	CAD (UA) vs Non-CAD (Adjusted) miR 21OR 2.488 95% CI (1.173, 5.277) p=<0.017 miR 25 OR 2.036 95% CI (1.048, 3.955) p =<0.036 miR 92a OR 2.611 95% CI (1.110, 6.144) p =< 0.028 miR 106b OR 2.389 95% CI (1.158, 4.927) p= <0.018 miR 126 OR 1.882 95% CI (1.140, 3.108) p =<0.013 miR 126 OR 1.882 95% CI (1.140, 3.108) p =<0.013 miR 451 OR 4.609 95% CI (2.171, 9.782) p =<0.001 miR 5905p OR 2.67895% CI (1.226, 5.849) p =<0.013	Adjusted for age, sex, HTN, dyslipidemia, DM, smoking status, and the use of statins and anti-platelet drugs)
9	Guo, et al., 2012 [19]	miR 122 miR 370	Up-regulated: miR 122 miR 370	QRT-PCR	Serum/ Plasma	HLD+CAD vs Non-CAD miR 122 OR 1.08 95 % CI 1.01–1.16 p = 0.034 miR 370 OR 1.05 95 % CI 1.01–1.12 p = 0.022	Adjustment for age, gender, BMI, smoking, HTN, DM, and blood lipid profiles
10	Sondermeijer, et al.,2011 [21]	miR 340 miR 624	Up-regulated: miR 340 miR 624	QRT-PCR	Platelets	Pre CAD vs Non-CAD miR-340/ miR624 95% CI: 0.59–0.83, p=<0.002	Univariate Analysis
11	Fichtlscherer, et al., 2010 [22]	miR 17 miR 92a miR 126 miR 133a miR 145 miR 155 miR 208a	Up-regulated: miR 133a miR 208a	QRT-PCR	Plasma/ Serum	CAD vs Non-CAD miR133a95% CI (5.22-6.35,3.94-6.10) p=0.16 miR208a 95% CI(5.72-6.74, 4.91-6.61) p=0.29	Univariate Analysis In patients with stable CAD, vascular-derivedMiRNAs were significantly down-regulated, whereas musclederivedMiRNAs tended to be higher.
12	Hokestra, et al., 2010 [24]	miR 135 miR 147	Up-regulated: miR 135	QRT-PCR	PBMCs	CAD vs Non-CAD miR 135a p=<0.001	Univariate Analysis
13	Takahashi, et al., 2010 [25]	miR146a/b	Up-regulated: miR146a/b	QRT-PCR	PMBC	CAD vs Non-CAD miR 146a/b p=<0.01	Adjusted for age, sex, culprit lesion, fasting glucose, HbA1C, LDL cholesterol, high-sensitive CRP, and history of HTN, DM, and corrected CAD. Marked TLR4 expression in atherosclerotic plaques, oxidative stress upregulates macrophage TLR4 expression, perhaps an association between TLR4, inflammation and coronary atherosclerosis. Activation of TLR4 signal may induce miR-146a/b expression as a negative regulator and induce progression of coronary atherosclerosis.
14	Li, et al., 2010 [26]	miR 21 miR 27b miR 130, miR 210	Up-regulated: miR 21 miR 27b miR 130 miR 210	QRT-PCR	Vessel Intima and Serum	AO vs Non-AO miR 21/27b/130/210 p=<0.05	Univariate Analysis
15	Minami, et al., 2009 [27]	miR 221 miR 222	Up-regulated: miR 221 miR 222	QRT-PCR	EP	Levels of miR 221 and miR 222 were higher in CAD group than in non-CAD group (p<0.01)	Univariate Analysis

Various different microRNA subtypes were studied with overlapping of the similar subtypes across the studies. Sayed, et al. did two different studies in two different age groups namely 40-60 and geriatric population. MicroRNA 756 was significantly up-regulated in both the middle-aged and elderly with a potential to be considered as a non-invasive biomarker in this population. Regulation of microRNA 223 was studied by Liu, et al. in 2015 and Diehl, et al. in 2012 in two case-control studies in China. Both the studies were not able to find a significant relationship between CAD and MiR 223, respectively. MiR 21 upregulation was tested in three of the studies: by Ren, et al. 2013, Diehl, et al., 2012 and Li, et al., 2010. Ren, et al. reported that the relationship of MiR 21 to CAD had the OR 2.488 CI 95% (1.173-5.277) p =0.017, while Diehl, et al. reported it to be p =0.042 and Li, et al. reported it to be p =0.05. MiR 92a and 126 were under study by two authors, namely, Fichtlscherer, et al. (2010) and Ren, et al. (2013). Ren, et al. reported that miR 92a and 126 when compared with CAD and non-CAD subjects had the OR 2.611; CI 95% (1.110-6.144) p =0.028 and OR 1.882;CI 95% (1.140-3.108) p =0.013, respectively. Fichtlscherer, et al. reported MiR 92a and 126 up-regulation had no significant relationship with CAD when compared with non-CAD patients. MiR 146a was also studied by two authors Takahashi, et al. and Diehl, et al. Both the authors were able to find a relationship of the up-regulation of miR 146a. Lastly, miRNA 155 was also studied by two authors, namely, Fichtlscherer, et al. and Diehl, et al. Neither studies found conclusive results for the significance of this biomarker with CAD subjects when compared with non-CAD subjects.

### Down-regulation and mircoRNA

A total of seven studies worked on the down-regulation of microRNA. All of the studies employed QRT-PCR as the method of analysis for detection of microRNA. Three studies used plasma as the source. Peripheral blood mononuclear cells were used by two studies. Two studies used whole blood as the source and one study used pericardial fluid and coronary arteries intima. All the studies reported down-regulation of microRNA in patients with CAD compared with non-CAD patients. The adjusted parameters were same as the up-regulation studies used.

MicroRNA subtypes which overlapped in the reviewed studies are miR 149, miR 155, and miR 145. MicroRNA 149 was studied by Sayed, et al. in 2015, in two different studies on two different age groups, which were 40-60 and a geriatric population. MicroRNA 149 was found to be down-regulated in both middle-aged and the geriatric patients. MicroRNA 155 was studied by four different authors namely, Hao, et al. 2014, Zhu, et al. 2014, Weber, et al. 2011 and Fichtlscherer, et al. Hao, et al. [28] reported 95% CI (6.92±0.93, 5.08±1.55) p =0.017, Zhu, et al. reported by Spearmen correlation analysis r =-0.663, p <0.001. Weber, et al. reported relation to be significant with the p =0.002 while Fichtlscherer, et al. reported that there was no significant relationship of microRNA 155 to CAD when compared to non-CAD patients (Table 3).

**Table 3:** Down-Regulated microRNA studies Reg: Regulated, QRT-PCR: Quantitative Reverse Transcription Polymerase Chain Reaction, CAD: Coronary Artery Disease, DM: Diabetes Mellitus, HTN: Hypertension, TC: Total Cholesterol, TAG: Triacylglycerols, HDL-c: High Density Lipoprotein Cholesterol, LDL-c: Low Density Lipoprotein Cholesterol, AST: Aspartate Aminotransferase, ALT: Alanine Transaminase, LVEF: Left Ventricle Ejection Fraction, CK-MB: Creatine Kinase for Myocardial B fraction, AP: Atherosclerotic Plaque, PBMC: Peripheral Blood Mononuclear Cells, RF: Risk Factor, CRP: C-Reactive Protein, ACEI: Angiotensin Converting Enzyme Inhibitor

	Name of Author; Year of Study	MicroRNA (miR or miRNA)	MicroRNA: Regulation	MicroRNA Analysis	Source	Strength of Association (Odds ratio, Relative Risk or Regression Analysis)	Comments
1.	Sayed, et al., 2015 [11]	miR149 miR 424 miR 765	Down Reg: miR 149 miR 424	QRT-PCR	Serum/ Plasma	Non-CAD vs. CAD (Adjusted) miR149 95% CI ( 0.894-0.983) p=0.0001 miR 424 95% CI (0.863-0.975) p=0.0001 miR 765 95% CI (0.939-0.996) p=0.0001	Adjusted for age, gender, TC, TG, HDL-C, LDL-C, systolic blood pressure, diastolic blood pressure, AST, ALT, creatinine, LVEF, DM, smoking, HTN and medications.
2.	Sayed, et al., 2015 [12]	miR149 miR 765	Down Reg: miR 149	QRT-PCR	Serum/ Plasma	CAD vs Non CAD (Adjusted) miR 149 p=0.001	Adjusted for subjects with similar age, gender, total cholesterol, total glyceride, HDL, LDL, systolic blood pressure, diastolic blood pressure, AST, ALT, creatinine, cardiac troponinI, CK-MB, LDH, LVEF, DM, smoking, HTN, and medications.
3.	Zhu, et al., 2014 [16]	miRNA 155	Down Reg: miR 155	QRT-PCR	PBMC	Correlation of miR-155 levels in PBMCs to Gensini scores in all patients (n=110). Spearman correlation analysis showed a negative correlation between miR-155 expression and the Gensini score in all patients; r = –0.663, p=0.001.	Adjusted ( miR-155 was correlated to multiple metabolic and CAD RF, including age, HTN, TC, HDL-C, LDL-C, Smoking, ACEI, statins, and CRP, but not sex, hereditary and DM or Impaired Glucose Tolerance.
4.	Weber, et al., 2011 [20]	miR 19a miR 29a miR 30e/5p miR 145 miR 150 miR 155 miR 181d miR 222 miR 342 miR 378 miR 584	Down Reg : miR 19a miR 29a miR 30e/5p miR 145 miR 150 miR 155 miR 181d miR 222 miR 342 miR 378 miR 584	QRT-PCR	Whole blood	CAD vs Non-CAD miR 19a p= 0.012 miR 29a p=0.012 miR 30e/5p p= 0.02 miR 145 p= 0.008 miR 150 p= 0.006 miR 155 p= 0.002 miR 222 p= 0.001 miR 342 p=0.001 miR 378 p= 0.001 miR 584 p= 0.036	Univariate Analysis As whole blood samples were studied, thus miRNA profile likely reflects intracellular and extracellular miRNAs levels, in contrast to exclusively extracellular miRNAs that would be detected in plasma.
5	Fichtlscherer, et al., 2010 [22]	miR 17 miR 92a miR 126 miR 133a miR 145 miR 155 miR 208a	Down Reg: miR 17 miR 92a miR 126 miR 145 miR 155	QRT-PCR	Plasma/ Serum	CAD vs Non-CAD miR 17 95% CI(9.06-10.97,5.42-8.29) p=0.001 miR 92a 95% CI (9.48-11.30,7.07-9.75) p= 0.01 miR 126 95% CI (9.86-12.02, 6.21-9.03) p=0.001 miR 145 95% CI (3.87-5.57, 4.81-5.90) p= 0.16	Univariate Analysis In patients with stable coronary artery disease, vascular-derived miRNAs were significantly down-regulated, whereas muscle derived miRNAs tended to be higher.
6	Taurino, et al., 2010 [23]	miR140/3p miR182	Down Reg : miR 140/3p miR 182 miR 92a/b	QRT-PCR	Whole blood	CAD vs Non-CAD miR-140-3p control vs. CAD p=0.002 miR-182 control vs. CAD p= 0.001 miR-92a/b control vs. CAD p=0.01	Univariate Analysis
7	Hoesktra, et al., 2010 [24]	miR 135 miR 147	Down Reg: miR 147	QRT-PCR	PBMC	CAD vs Non-CAD miR 147 p=<0.01	Univariate Analysis

## Discussion

MiRNAs have been known to have an association with the physiological and pathological processes involved in the development of CADs such as endothelial dysfunction, inflammation, apoptosis, angiogenesis, atherosclerosis, and neointimal hyperplasia or restenosis [7,29-31]. Our systematic review summarizes 18 articles comparing miRNAs in CAD patients. Ten studies found miRNAs that were up-regulated (21-3135b), five studies showed miRNAs that were both up-regulated (21-765) and down-regulated (17-222), and three studies showed down-regulated miRNAs (19a-584). miRNAs derived from plasma/serum, microparticles, whole blood, platelets, blood mononuclear intimal and endothelial progenitor cells were investigated. Circulating miRNAs (plasma or serum) exhibit remarkable stability and are highly resistant to plasma ribonuclease (RNase) activity due to internalization in macro vesicles and the formation of protein-miRNA complexes and can be used as a reliable marker for both the early diagnosis and prognosis of CAD. Therefore, the levels of individual cardiac-enriched circulating miRNAs are related to the diagnosis and prognosis of heart diseases. Recent studies have shown the involvement of miRNAs in atherosclerosis, ranging from endothelial dysfunction to plaque rupture suggesting the use of miRNA as potential biomarkers in early diagnosis of CAD [32]. miRNA upregulation or downregulation is either related to the atherosclerotic disease process or the inflammatory compensation [21].

Sayed, et al. in elderly and Zhu, et al in middle-aged documented that miRNAs (149, 424 and 155, respectively) were down-regulated in CAD patients, Zhu, et al. postulated that miRNA 155 was downregulated because of the feedback mechanism that controls the overactivation of immune cells and thus it is negatively correlated with coronary stenosis. Similarly, Fichtlscherer, et al. found miRNA 155 to be downregulated in patients with CAD. Fichtlscherer suggested that atherosclerotic lesions uptake circulating miRNAs, thereby decreasing their levels in circulation and causing downregulation. miRNA 155 is noteworthy as it was found to be downregulated in multiple studies which used different sources (serum plasma, whole blood, and peripheral blood mononuclear cells (PMBC). Previously, Sondermeijer, et al. have reported miRNA 624 and 340 to be down-regulated in the premature CAD patients [33].

Sayed, et al., Hoekstra, et al., and Ren, et al. showed an up-regulation of miRNA 765, 134-370 and 21-5905p, respectively, in the middle-aged population of unstable CAD patients as well. Ren, et al. stated that miRNA (21-5905p) that are involved in the pathogenesis of vulnerable plaque are mostly unregulated. In these patient populations, miRNA can be used as novel biomarkers in the diagnosis of disease. The severity of the disease can be potentially seen by aberrancy of miRNA in form of either up-regulation or down-regulation. Prior studies have shown that the levels of miRNA are linked with disease severity. The up-regulated miRNA leads to the evolution of plaque towards growth, instability, and rupture [33]. Fichtlscherer, et al. in a prospective case control trial studied miRNA derived from endothelial, cardiac, skeletal, and smooth muscle cells. Interestingly, in CAD patients endothelial and smooth muscle miRNAs were up-regulated and in cardiac and skeletal muscle cells miRNA 133a/208a/155 were down-regulated in the same patient population. Prior studies have also documented the differential role of miRNA in skeletal, smooth and cardiac muscle [34]. The reason for this asymmetric regulation is an uptake of miRNAs in cardiac muscles by atherosclerotic lesions in apoptotic bodies and endothelial cells are exposed to inflammatory cells that can increase miRNA due to cellular stressors (Fichtlscherer, et al). miRNA can be specifically monitored in certain disease processes and used as prognostic markers as seen in CAD patients with diabetes, miRNA 145 being reduced compared to those without DM (Fichtlscherer, et al). For instance, Sondermeijer, et al. showed that the platelet-derived miRNA can potentially fine-tune the expression of specific gene products that may be involved in governing platelet reactivity. Therefore, a dysfunctional miRNA-based regulatory system could lead to the development of serious platelet-related cardiovascular diseases (Sondermeijer, et al).

Studies by Zhou, et al. and Li, et al. in CAD patients reported an upregulation of plasma-derived miRNA (140-3p/182, 206/574-5p, 134-3135b respectively). Taurino, et al. showed downregulation of whole blood-derived miRNA 92-182 at the genetic level. Interestingly, Guo, et al. and Minami, et al. in statin therapy CAD patients documented a significant downregulation of miRNA 122/370 and 221/222 levels that were previously up-regulated. Endothelial progenitor cells play an important level and in the regulation of miRNA after treatment with lipid therapy on lipometabolism related miRNA (Minami, et al.). Similarly, Takahashi, et al. noted a decrease in the miRNA 146 a-b levels in CAD patients in post-statin and angiotensin-converting enzyme inhibitor/angiotensin receptor blocker (ACEI/ARB) therapy as well. In a cross-sectional study by Weber, et al., miRNA 19a-584 was also reported to be significantly down-regulated in CAD patients post ACE/ARB therapy. The response of miRNA to medications may provide an insight into the prognostic value of miRNA. Pharmacological therapy has been known to have an effect through gene signaling pathway, which further influences miRNA levels [7]. Diehl, et al. examined microparticles derived miRNA 19-223 and documented an upregulation in acute coronary syndrome (ACS) patients. Certain miRNA are shown to be involved in cardiac hypertrophy and angiogenesis and shown to be up-regulated in microparticles (Diehl, et al). Interestingly, Li, et al. observed upregulation of miRNA 21-210 in the intima of arteriosclerosis obliterans (ASO) patients. miRNA has been shown to be pro-angiogenic through its effects at the genetic level (Li, et al). 

### Limitations

Firstly, criteria for CAD participant enrolment across the studies were not standardized. Secondly, the study cohorts were not age and sex matched. Thirdly, the population size of different trials was very small. Fourthly, none of the included studies prospectively analyzed a specific miRNA regulation derived from one source or from different sources. Even specific miRNA regulation can be varied when compared across different studies and sources of origin. The pre and post CAD miRNA levels were not mentioned by any of the studies. Large randomized double-blinded studies are needed to eliminate population bias and increase the statistical power of each study. Prospective case-control trials with longer follow-up periods may play a role in deciphering the prognostic potential for the miRNA markers in CAD patients. Lastly, studies included did not mention specifically the number of patients on ACE/ARBs or statins.

## Conclusions

miRNA expression profile is associated with several human cardiovascular diseases, suggesting their role as a novel class of biomarkers as well as potential treatment targets for cardiovascular diseases. This systematic review provides a potential insight on miRNA regulations in subjects with and without CAD and highlights the different parameters which could be the reason for aberrant miRNA expression. Further understanding of miRNA expression may help to delineate their role in improving both the diagnostic and therapeutic approaches to stratifying CAD burden in the general population.
